# The Economic Impact of COVID-19 Treatment at a Hospital-level: Investment and Financial Registers of Brazilian Hospitals

**DOI:** 10.36469/jheor.2021.22066

**Published:** 2021-04-16

**Authors:** Ana Paula Beck da Silva Etges, Ricardo Bertoglio Cardoso, Milena S Marcolino, Karen Brasil Ruschel, Ana Paula Coutinho, Elayne Crestani Pereira, Fernando Anschau, Fernando Aranha, Filipe Carrilho, Giovanna Vietta, Gisele Alsina Nader Bastos, Joanna d’Arc Lyra Batista, José Miguel Chatkin, Juliana da Silva Nogueira, Leila Beltrami Moreira, Luciana Haddad, Maiara Floriani, Marília Teixeira de Siqueira, Patrícia Ziegelmann, Petrônio José de Lima Martelli, Roberta Pozza, Umbelina Cravo Teixeira Lagioia, Carisi Anne Polanczyk, Luciane Kopittke

**Affiliations:** 1 National Institute of Science and Technology for Health Technology Assessment (IATS), Porto Alegre, Rio Grande do Sul, Brazil; Graduate Program in Epidemiology, Universidade Federal do Rio Grande do Sul, Brazil; School of Technology, Pontifícia Universidade Católica do Rio Grande do Sul, Porto Alegre, Brazil; 2 Graduate Program in Epidemiology, Universidade Federal do Rio Grande do Sul, Brazil; 3 Department of Internal Medicine, Medical School; Telehealth Center, University hospital; Universidade Federal de Minas Gerais, Belo Horizonte, Brazil; 4 National Institute of Science and Technology for Health Technology Assessment (IATS), Porto Alegre, Rio Grande do Sul, Brazil; 5 Hospital de Clínicas de Porto Alegre, Brazil; 6 Hospital SOS Cárdio de Florianópolis, Brazil; 7 Grupo Hospital Nossa Senhora da Conceição, Brazil; 8 Hospital das Clínicas da Universidade Federal de Pernambuco, Brazil; 9 Hospital Moinhos de Vento, Brazil; 10 Universidade Federal da Fronteira Sul, Brazil; 11 Hospital São Lucas da Pontifícia Universidade Católica do Rio Grande do Sul, Brazil; 12 Universidade Federal de Pernambuco, Brazil; 13 Graduate Program in Epidemiology, Universidade Federal do Rio Grande do Sul, Brazil; Hospital de Clínicas de Porto Alegre, Brazil; 14 Hospital das Clínicas da Faculdade de Medicina Universidade de São Paulo, Brazil; 15 Pronto Socorro Cardiológico Universitário de Pernambuco (PROCAPE), Brazil; 16 Hospital Tacchini, Brazil; 17 National Institute of Science and Technology for Health Technology Assessment (IATS), Porto Alegre, Rio Grande do Sul, Brazil; Graduate Program in Epidemiology, Universidade Federal do Rio Grande do Sul, Brazil; Graduate Program In Cardiology, Universidade Federal do Rio Grande do Sul, Brazil

**Keywords:** hospital management, covid-19 investment, value-based health care, hospital costs, economic analysis, covid-19

## Abstract

**Background:** The economic impact associated with the treatment strategies of coronavirus disease-2019 (COVID-19) patients by hospitals and health-care systems in Brazil is unknown and difficult to estimate. This research describes the investments made to absorb the demand for treatment and the changes in occupation rates and billing in Brazilian hospitals.

**Methods:** This research covers the initial findings of “COVID-19 hospital costs and the proposition of a bundled reimbursement strategy for the health-care system,” which includes 10 hospitals. The chief financial officer, the chief medical officer, and hospital executives of each participating hospital provided information regarding investments attributed to COVID-19 patient treatment. The analysis included variations in occupation rates and billing from 2019 to 2020 observed in each institution, and the investments for medical equipment, individual protection materials and building construction per patient treated.

**Results:** The majority of hospitals registered a decrease in hospitalization rates and revenue from 2019 to 2020. For intensive care units (ICUs), the mean occupancy rate ranged from 88% to 83%, and for wards, it ranged from 85% to 73%. Monthly average revenue decreased by 10%. The mean hospital investment per COVID-19 inpatient was I6800(standarddeviation7664),withthepurchaseofventilatorsasthemostcommoninvestment.Forthisitem,themean,highestandlowestacquisitioncostperventilatorwere,respectively,I31 468, I48 881andI17 777.

**Conclusion:** There was significant variability in acquisition costs and investments by institution for responding to the COVID-19 pandemic. These findings highlight the importance of continuing microeconomic studies for a comprehensive assessment of hospital costs. Only with more detailed analyses, will it be possible to define and drive sustainable strategies to manage and reimburse COVID-19 treatment in health-care systems.

## INTRODUCTION

One of the greatest concerns regarding the coronavirus disease-2019 (COVID-19) pandemic is the disease’s burden on the health-care system.^1^ The necessity of general and effective organization of health-care services to safely treat the population worldwide and to fight against the virus required a massive effort to reallocate health-care professionals, provide proper medications, produce a vaccine, and deliver the needed increase in capacity.^2^

The initial restricted understanding of the disease’s life cycle led hospitals and health-care services to pursue different internal management strategies to meet the demand for COVID-19 patient care.^3^ Several papers were published on these strategies in different countries, for example, cases from Mount Sinai hospitals in New York, NY,^3^ a private hospital in Brazil,^4^ a general hospital in Naning, China^5^ and primary care redesigns.^6^ A converging aspect on how hospitals approached the pandemic included the need for relocating and hiring health professionals, improving communication between teams and having agile and integrated care. Berwick DM used the expression “choices for the new normal” to point out 6 properties of care for durable change: tempo, standards, working conditions, proximity, preparedness, and equity. For the author, the care modifications required by the pandemic can enable health-care systems to become more patient-centered, efficient and, consequently, able to deliver better care to the population.^7^

Similarly, a few specialists have discussed how the modifications established to prepare the health-care system to treat COVID-19 patients could contribute to accelerating the process of driving health-care value.^2,8^ In a value-driven health-care system, which aims to deliver care with quality for the majority of the population, the search for waste reduction is continuous.^9^ The 2020 COVID-19 pandemic brought to light the need for health systems to have more effective care delivery processes. Developing an integrated health system is elementary to migrate the health system from volume to value. The pandemic has accelerated the implementation of aligned strategies for providers, payers, policymakers, employers, and patients, which can help seize the opportunity to build a better and connected health system.^10^ Schaye et al. (2020) described a successful experience in New York City, in which a planned collaboration across private, public and academic hospitals achieved good results through the implementation of successful strategies for communication, surge planning, clinical care and staff wellness.^11^

Implementing those strategies in a short length of time to support a global issue required a large number of investments in the health-care system worldwide. The total economic impact associated with the strategies assumed by the hospitals and health-care systems are unknown and difficult to estimate yet, but it can be divided in investments to prepare organizations to treat COVID-19 patients^2^; and decreases in revenue, due, for example, to canceling or postponing elective surgeries and procedures.^12,13^

In Brazil, the impact of the pandemic has been devastating. Through February 23, 2021, there were over 10.3 million confirmed cases and approximately 250 000 reported deaths. These numbers are probably underestimated, according to the Brazilian Ministry of Health’s real-time counting system (Painel Coronavírus: https://covid.saude.gov.br/). The “auxilio emergencial,” the Brazilian national assistance program introduced by the Ministry of Economy to support low-income families, called for a direct investment of I$142 billion (R$321 billion—almost 4% of Brazilian GDP). The macroeconomic information is being continuously updated.^14^ Therefore, the pandemic’s economic impact at the health-care organization level is still unknown in Brazil.

To provide an overview of the economic impact of the first 10 months of the COVID-19 pandemic in Brazil at a hospital level, the objective of this study was to describe the investments made to absorb the demand for treatment and the changes in occupancy rates and revenues, in comparison to the year before the pandemic.

## METHODS

This report covers the initial findings of the research project: “COVID-19 hospital costs and the proposition of a bundled reimbursement strategy for the health-care system” (CAAE: 30350820·5·1001·0008), which aims to identify the actual hospital COVID-19 patient treatment cost information to scientifically subsidize reimbursement strategies in a bundled model.

All the centers participating at the national COVID-19 clinical registry research (N=25 hospitals) where invited to collaborate into the cost study.^15^ A total of 10 hospitals accepted the invitation and were included in this research. The hospitals were from 4 Brazilian states (Rio Grande do Sul, Santa Catarina, São Paulo, and Pernambuco) in the South, Southeast, and Northeast regions, and treated high- and low-complexity COVID-19 patients. All patients admitted for COVID-19 treatment from the beginning of the pandemic in Brazil, from March 2020 through September 2020, in whom the disease was confirmed by the reverse transcription–polymerase chain reaction or serological tests, according to the World Health Organization criteria,^16^ were considered in the analysis.

The chief financial officer, the chief medical officer and hospital executives at each participating hospital provided information regarding investments that to their knowledge were attributed to COVID-19 patient treatment. A standardized instrument was available for each chief financial officer to guide the report of the economic data, which is available as supplementary material (Table S1). To reduce the risk of bias, instructions about how to use the instrument were provided in a videoconference training meeting and whenever possible accounting reports and fiscal billings were shared by each hospital center to review the accuracy of the data collected.

Cases in which acquisition of medical equipment was already in the budget for 2020 were not considered COVID-19 specific investments. For example, if one hospital was planning an acquisition of medical equipment for 2020, this investment was not considered a COVID-19 investment. The collected data and analysis included the variations in occupancy rates (ward and intensive care unit [ICU]), revenues from 2019 to 2020 observed in each institution, and the investments in medical equipment, individual protection materials and building construction per patient treated. While the mean values from 2019 considered the full year, the mean values from 2020 only covered the period from the beginning of the pandemic, March 2020, through the time limit of the data collection of this report, September 2020.

The analyses were descriptive, and in a database developed in Microsoft Excel for Mac 2021 (Palo Alto, USA). The financial data were collected in Brazilian currency (*Reais*, in 2020) and converted into international dollars according to the purchasing power parity value (I$, in 2019).^17^

## RESULTS

Hospitals classified as a regional reference center for COVID-19 treated more patients than the others included in our sample, and it appears that those hospitals treated patients with more severe clinical conditions. From the state, Rio Grande do Sul, hospitals’ sample represented over 50% of the total amount hospitalized for COVID-19 in this region. Table 1 describes characteristics of the participating hospitals.

**Table 1:** Characteristics of Participating Hospitals

**Hospital**	**Profile**	**Brazilian State**	Regional Reference for COVID-19*	COVID-19 Patients Hospitalized**	**Total Beds**	COVID-19 ICU Beds***	COVID-19 Ward Beds***
A	Public and academic	Rio Grande do Sul	Yes	1232	831	105	82
B	Public and academic	Rio Grande do Sul	Yes	1082	796	44	82
C	Public and academic	São Paulo	Yes	2000	918	300	300
D	Public and academic	Pernambuco	No	145	411	18	18
E	Private	Rio Grande do Sul	Yes	400	568	40	42
F	Public	Pernambuco	No	150	256	18	18
G	Public	Santa Catarina	No	363	339	*40*	*59*
H	Private	Rio Grande do Sul	Yes	836	485	57	88
I	Private	Santa Catarina	No	229	113	32	18
J	Private/public/ academic	Rio Grande do Sul	No	350	416	59	134

Abbreviations: ICU; intensive care unit.

* It is estimated that more severe patients were treated at regional reference centers.

** From the first patient, March 2020 until September 2020.

*** At the peak of the COVID-19 pandemic in 2020.

The majority of hospitals registered a decrease in the ICU occupancy rate from 2019 to the period of 2020 considered (Table 2) and only one hospital registered an increase in the ICU occupancy rate. For the ICU, the mean occupancy rate ranged from 88% to 83%. Regarding general ward occupancy rates, the differences observed were higher than for the ICU. At six hospitals, there was an absolute decrease greater than 10 percentage points. Only one hospital registered an increase in the occupancy rate, which was a private hospital. Mean ward hospitalization rates ranged from 85% to 73%, and the general mean (ward and ICU) hospitalizations ranged from 86% to 78%. The parameter observed for 2020 was similar to the annual report from the private health sector in Brazil in 2018, which was 76% (75% at the South: hospitals A, B, E, G, H, I and J; 77% at the Southeast: hospital C; and 74% at the Northeast: hospitals D and F).^18^

**Table 2:** Hospital Occupancy Rate Variation from 2019 to 2020

**Hospital**	**ICU Occupancy Rate**		**Variation (PP)**	**Hospital Occupancy Rate**		**Variation (PP)**
	**2019**	**2020**		**2019**	**2020**	
**A**	85%	74%	-11	85%	70%	-14
**B**	91%	86%	-5	85 %	69%	-16
**C**	95%	94%	-0.9	80%	78%	-2
**D**	88%	118%	30	80%	77%	-3
**E**	97%	86%	-11	80%	63%	-17
**F**	69%	57%	-12	100%	66%	-34
**G**	97%	96%	-1	84%	72%	-11
**H**	79%	76%	-3	87%	92%	4
**I**	95%	78%	-16	82%	79%	-2
**J**	84%	72%	-11	83%	66%	-16

Abbreviations: PP; percentage points.

The case mix from diagnosis related group (DRG) codes at the hospital that registered an increase in the ward hospitalization occupancy rate (H) varied from 1.30 to 1.34. This hospital registered a 22% decrease in its mean income per month. The remaining institutions do not code DRGs, thus this information was not available. However, it was possible to analyze the impact of COVID-19 on the hospitals’ revenue. In the same period of both years, the majority of hospitals registered a decrease in the mean income per month, which decreased by 10% on average.

The mean investment per COVID-19 patient was I$6800 (SD 7664). Table 3 contains the investment breakdown of each hospital. In general, acquisition of mechanical ventilators was the most common investment attributed to COVID-19 management. For this specific item, there was a high variability in the unitary acquisition cost among hospitals. The mean, highest and lowest acquisition cost per ventilator were, respectively, I$31 468, I$48 881 and I$17 777. The highest prices were paid by hospitals that bought more ventilators and had a higher concentration of COVID-19 patients (Table S2). Regarding individual protection equipment, the lowest investment was registered by two of the private hospitals.

**Table 3:** Investment for COVID-19 Management Preparedness per Patient Assisted

**Hospital**	**Mechanical Ventilators**	**Building Engineering**	**Medical Equipment**	**Individual Protection Equipment**	**Total Invested per Patient**
**A**	I$3307 (33%)	-	I$6422 (65%)	I$159 (2%)	I$9889
**B**	I$1155 (25%)	-	-	I$3531 (75%)	I$4687
**C**	I$4836 (74%)	I$180 (3%)	I$236 (4%)	I$1254 (19%)	I$6505
**D**	I$1692 (6%)	I$1974 (7%)	I$316 (1%)	I$23 492 (86%)	I$27 474
**E**	I$380 (13%)	I$326 (11%)	I$2195 (75%)	I$44 (1%)	I$2945
**F**	I$474 (34%)	-	I$916 (66%)	-	I$1390
**G**	I$2477 (51%)	I$34 (1%)	I$1112 (23%)	I$1262 (26%)	I$4885
**H**	I$843 (26%)	I$213 (7%)	I$2124 (67%)	I$8 (0.25%)	I$3188
**I**	-	-	-	I$1954 (100%)	I$1954
**J**	I$1384 (27%)	I$13 (0.2%)	-	I$3689 (73%)	I$5086

All the financial information reported is considering the full period of the study, March 2020 until September 2020.

The percentage indicates the proportion of investment for each item.

Hospital F reported investment in Individual Protection Equipment, but the hospital was not able to inform the total amount specified for this item.

## DISCUSSION

This research presents the characteristics of hospitals from different regions of Brazil, a comparison of demand for health-care services between the pandemic and non-pandemic periods, hospitals’ capacity to manage COVID-19 inpatients and the respective investment strategies to respond to the pandemic demands. The results demonstrated that the majority of hospital investments focused on the purchase of mechanical ventilators, and that financial investments added to decreases in the occupancy rates indicating the significant direct and indirect economic impact of the pandemic at the hospital level.

Financial sustainability has to be constantly addressed by hospitals. In this context, the occurrence of a natural, catastrophic event such as the COVID-19 pandemic is recognized by health-care leaders as a relevant enterprise financial risk, which deserves continuous vigilance from leadership to avoid larger economic impacts.^19^ Although recent studies in Brazil have shown that hospitals that provide inpatient care for the public health system are inefficient and should work to increase production and reduce inputs to achieve a better economic sustainability,^20^ this research demonstrates an opposite behavior during the COVID-19 pandemic: a decrease in occupancy rates and revenues and an increase in investments, suggesting the importance of developing strategies to contribute to health organizations’ financial sustainability for the future. The cancelation or postponement of elective surgeries and procedures could have contributed to the decrease of revenue and occupation rates. According to the administrative database, DATASUS, from the Ministry of Health, the number of elective surgeries decreased by 34.7% across the country from 2019 to 2020.^21^

Achieving better performance in health-care organizations requires strategy aligning.^22^ By the speed at which the pandemic is advancing, health-care systems are being forced to redesign their work flows to be able to deliver care while seeking solutions to make it more efficient and economically-viable.^23^ Our results indicate that the investment strategies adopted by hospitals can diverge, but in all cases, organizational investments were necessary to support the demand for hospital services.

Notably, the organizational strategies are oriented to be prepared for treating a proportion of the population affected by the disease, but the hospitals also are suffering the economic impact caused by the pandemic in all business markets. The most frequent model to reimburse hospitals in Brazil is based on volume (fee-for-service [FFS]), which does not consider the quality of care and outcomes that are being delivered by the institutions.^24^ For the specific case of COVID-19, the reimbursement strategies varied among the institutions, but all the participating hospitals used a FFS strategy to reimburse COVID-19 care. Usually in this model, up-front medical equipment and safety supplies costs demonstrated in this report are difficult to be fully captured. It is also difficult to capture the indirect effect on other disease care. For instance, patients admitted for other medical conditions usually have their treatment delayed due to SARS-COV-2 infection screening, leading to longer length-of-stay and possibly worse outcomes for time-sensitive conditions.

Only one participating hospital used DRG codes, which exemplified the deficiency of a FFS reimbursement system, as it observed a decrease in the mean monthly income from 2019 to 2020, an increase in its ward occupancy rate, and an increase in its mean DRG case mix (1.30-1.34). A study that used data from Medicare and commercial claims sources in the US for hospitalization due to influenza/viral pneumonia to estimate the potential cost-savings of reducing ICU length of stay demonstrated that the average cost per additional day of ICU hospitalization would be within the range of I$3900-I$5646 for commercially insured patients; for Medicare-FFS patients, ICU stays would be within a range of I$635-I$1150. Cost information was based on how much was paid by the insurance companies or Medicare. One reason the authors gave for the observed difference was that, in Medicare, this cost represented fixed pricing based on DRGs, with the same reimbursement regardless of length of stay.^25^ While the studies do not advance for comprehending the cost to deliver care to patients instead of comparing how much it paid, the health-care system will continue to look for opportunities to reduce waste and increase the capability to deliver high-quality care.

## CONCLUSION

Few authors have discussed in the literature why COVID-19 can accelerate redesigning the health-care system to become more efficient, proactive, sustainable, and patient-centered.^23,26^ To achieve this redesign and deliver those outcomes, it is crucial to implement an accurate capability to measure and control costs and clinical outcomes and adopt reimbursement strategies that will maintain the health system structure.^27^ The high variability observed for the acquisition costs and investments in each institution observed in this research highlights the importance of continuing the microeconomic studies of the COVID-19 pandemic, such as the hospital costs linked to patients’ comorbidities and hospital technologies. With a more detailed understanding of costs per COVID-19 patient, it will be possible to define and drive sustainable strategies to manage and reimburse COVID-19 treatment in health-care systems.

### Limitations and Future Studies

The inclusion of hospitals from one country, which were not randomly selected, and the small sample of hospitals are limitations of this research, which require caution while generalizing the results to other settings. For the occupation rates comparisons between 2019 and 2020, the use of the full year for 2019 and only the period from March to September for 2020 is understood as a limitation of the present study and a future analysis including the complete year of 2020 is strongly suggested. The decrease in occupation rates findings could be associated with the preventive strategy adopted of canceling or postponing elective surgeries and procedures.^12,13^ However, specific analyses could not be done to confirm this hypothesis.

In addition, the information obtained was not valued by the quality or characteristics of investments done or by the level of clinical severity of the patients treated in each hospital. At this moment, it is being consolidated in a report of the economic impact caused by the COVID-19 disease treatment at the hospital level, and the variability observed reinforces the importance of the continuity of the study to understand costs at a patient level, including the variability in clinical conditions among patients.

## Supplementary Material

Supplementary Material
