# Supplementary material for: The Economic Impact of COVID-19 Treatment at a Hospital-level: Investment and Financial Registers of Brazilian Hospitals
Source: J Health Econ Outcomes Res. 2021 Apr 16;8(1):36–41. doi: 10.36469/jheor.2021.22066 (PMC8051953; doi:10.36469/jheor.2021.22066)
Supplement: Supplementary Material [file jheor_2021_8_1_22066_57211.pdf]

Journal of Health Economics and Outcomes Research

Supplementary Online Material

Beck da Silva Etges AP, Cardoso RB, Marcolino M, *et al*. The economic impact of COVID-19 treatment at a hospital-level: investment and financial registers of Brazilian hospitals. *JHEOR*. 2021;8(1):36-41.
doi:10.36469/jheor.2021.22066

Table S1. Institutional COVID-19 Costs
Table S2. Cost of Acquisition of Mechanical Ventilators

This supplementary material has been provided by the authors to give readers additional information about their work.

This is an open-access article distributed under the terms of the Creative Commons Attribution 4.0 International License (CCBY-4.0). View this license's legal deed at http://creativecommons.org/licenses/by/4.0 and legal code at http://creativecommons.org/licenses/by/4.0/legalcode for more information.

Table S1. Institutional COVID-19 Costs

https://drive.google.com/file/d/1b74MFN7udaCkPGLOmaxrHlV03NLojfa4/view?usp=sharing

Table S2. Cost of Acquisition of Mechanical Ventilators

Hospitals	Ventilators Unitary Acquisition Cost	Number of Ventilators Aquired
**A**	I$45 777	89
**B**	I$48 881	35
**C**	I$32 783	295
**D**	I$20 440	12
**E**	I$30 476	5
**F**	I$17 777	4
**G**	I$26 444	34
**H**	I$27 111	26
**I**	-	0
**J**	I$28 496	17
